# Digitally Enabled Peer Support and Social Health Platform for Vulnerable Adults With Loneliness and Symptomatic Mental Illness: Cohort Analysis

**DOI:** 10.2196/58263

**Published:** 2024-07-24

**Authors:** Dena Bravata, Daniel Russell, Annette Fellows, Ron Goldman, Elizabeth Pace

**Affiliations:** 1 Center for Primary Care and Outcomes Research Stanford University San Mateo, CA United States; 2 Wisdo Health Inc New York, NY United States; 3 Human Development and Family Studies Iowa State University Ames, IA United States; 4 Foundation for Social Connection Washington DC, DC United States; 5 Department of Technology Management and Innovation Tandon School of Engineering New York University New York, NY United States; 6 Peer Assistance Services, Inc Denver, CO United States

**Keywords:** peer support, social isolation, loneliness, depression, depressive, mental health, anxiety, quality of life, isolation, isolated, online support, digital health, vulnerable, race, racial ethnic, ethnicity, gender, socioeconomic, demographic

## Abstract

This study prospectively evaluated the effects of digitally enabled peer support on mental health outcomes and estimated medical cost reductions among vulnerable adults with symptomatic depression, anxiety, and significant loneliness to address the mental health crisis in the United States.

## Introduction

In the United States, 50% of adults currently experience loneliness, with rates exceeding 75% for non-White populations and 72% for those on Medicaid [1]. The US Surgeon General’s advisory about the loneliness epidemic reported that poor social connection confers the same mortality risk as smoking 15 cigarettes a day and doubles the risk of depression and anxiety [1]. Annual health care spending is US $1644 higher for socially isolated individuals than for those who are not socially isolated [2].

Peer support is a promising intervention for adults with loneliness, depression, and anxiety [3]; however, digitally enabled peer support has not been evaluated for populations with severe loneliness or symptomatic mental illness. To fill this gap, this study prospectively evaluated (1) engagement with a digitally enabled peer support intervention; (2) changes in mental health outcomes; and (3) estimated health care cost reductions among vulnerable adults with symptomatic depression, anxiety, and significant loneliness.

## Methods

### Participants

Adults (>18 years) living in Colorado were recruited via social media campaigns between January 1 and December 31, 2022, to participate in a peer support program [3]. The digital intervention is an online peer support and social health platform offering ≥30 peer communities for members struggling with mental health, chronic conditions, life stressors, and social determinants of health. Using artificial intelligence, the platform proactively connects members with peers based on shared lived experiences to provide emotional support and companionship.

### Data Collection and Analysis

Participants completed surveys at baseline and at 30, 60, and 90 days into the intervention, which included validated questions from the UCLA-3 Loneliness Scale [4], 2-item Patient Health Questionnaire (PHQ-2) [5], 7-item General Anxiety Disorder (GAD-7) scale [6], and mentally unhealthy day scale [7]. Social vulnerability index scores were assigned according to the participants’ home census tract [8]. We estimated a reduction in annual medical costs from research demonstrating that each unhealthy day is associated with an incremental cost of US $15.64 per member per month [9].

To evaluate the role of peer support for individuals with symptomatic mental health illness, we included only participants with a baseline PHQ-2 score>4; UCLA-3 score>7; or those who reported feeling nervous, anxious, or on edge more than half the days in the prior 2 weeks.

Baseline mental health and loneliness outcomes were compared with those of the last survey at 30, 60, or 90 days using *t* tests. We used ANOVA to assess engagement and changes in clinical outcomes by age, race/ethnicity, and gender.

### Ethical Considerations

The study was approved by the WCG Institutional Review Board (Wisdo.001.1/26/2023). Since all data were routinely collected during the intervention, this protocol was considered exempt from additional consent. All data were deidentified. Participants received 1 year of free access to the platform but no other compensation.

## Results

Among 1104 enrollees, 243 reported significant loneliness and/or mental health symptoms and completed at least one follow-up survey, thereby qualifying for inclusion (Table 1). Engagement with peer support was high and similar across genders, races, and socioeconomic groups (*P*>.05 for all comparisons), with an average of 8 visits per month. Engagement rates with peer support were 84.4% in month 2 and 70.6% in month 3.

Participants of all genders, race/ethnicities, and socioeconomic groups experienced statistically and clinically significant (all *P*<.001) improvements in loneliness (11.1%), depression (28.3%), and number of mentally unhealthy days (26.2%). For example, Hispanic/Latinx participants reported 6.7% and 25.6% improvements in loneliness and depression, respectively, while Black/African American participants reported 15.7% and 27.3% improvements in loneliness and depression, respectively. The average reduction of 5.46 mentally unhealthy days at month 3 compared to baseline resulted in an estimated annual medical cost reduction of US $1025/participant. Participants who engaged with digital peer support at 90 days had the greatest improvements in their loneliness and mental health outcomes (Table 2).

**Table 1:** Baseline participant demographics (N=243).

Characteristic		Value
Age (years), mean (SD)		43.2 (14)
**Age group (years), n (%)**		
	18-29	35 (14.4)
	30-39	79 (32.1)
	40-49	49 (20.2)
	50-59	35 (14.4)
	60+	43 (17.7)
	No response	3 (1.2)
**Sex, n (%)**		
	Male	135 (55.7)
	Female	99 (40.6)
	Nonbinary	8 (3.4)
	No response	1 (0.4)
**Race/ethnicity, n (%)**		
	White	170 (69.7)
	Hispanic/Latinx	37 (15.4)
	Black/African American	20 (8.3)
	Other	16 (6.7)
**Social vulnerability index, n (%)^a^**		
	High (0.75-1)	25 (10.4)
	Medium (0.5-0.75)	158 (64.7)
	Medium-low (0.25-0.5)	49 (20.3)
	Low (0-0.25)	11 (4.6)
**PHQ-2^b^** **Depression Scale^c^**		
	Total score, mean (SD)	3.8 (1.8)
	0, n (%)	8 (3.3)
	1, n (%)	11 (4.6)
	2, n (%)	50 (20.7)
	3, n (%)	36 (14.7)
	4, n (%)	43 (17.8)
	5, n (%)	30 (12.4)
	6, n (%)	65 (26.7)
**UCLA-3 Loneliness Scale^d^**		
	Total score, mean (SD)	7.9 (1.3)
	3, n (%)	3 (1.2)
	4, n (%)	3 (1.2)
	5, n (%)	12 (5)
	6, n (%)	14 (5.6)
	7, n (%)	45 (18.6)
	8, n (%)	58 (24)
	9, n (%)	108 (44.4)
**GAD-7^e^** **Single Anxiety Item**		
	Total score, mean (SD)	1.9 (1)
	0 (no anxiety), n (%)	21 (8.7)
	1, n (%)	68 (27.9)
	2, n (%)	65 (26.9)
	3 (nearly every day), n (%)	89 (36.6)
**Mentally unhealthy days in past month**		
	Number of days, mean (SD)	21.3 (9.1)
	0-5, n (%)	22 (9.1)
	6-10, n (%)	17 (7)
	11-20, n (%)	76 (31.3)
	21-30, n (%)	125 (51.4)
	No response, n (%)	3 (1.2)

^a^The social vulnerability index is scored as a percentile from 0 (least vulnerable) to 1 (most vulnerable) that allows geographies to be directly compared based on 16 measures, including socioeconomic status (rate of population below 150% of the federal poverty line, unemployed, high housing cost burden, no high school diploma, no health insurance); household characteristics (percent 65 years or older, 17 years or younger, civilians with a disability, single-parent households, English-language proficiency); racial and ethnic minority status; and housing type and transportation (rate of multiunit structures, mobile homes, crowding, no vehicle, group quarters) [8]. Since we collected racial/ethnic group information from the participants directly, the total social variability index and three other index categories are reported.

^b^PHQ-2: 2-item Patient Health Questionnaire.

^c^Items on the Depression Scale are scored from 0 (not at all) to 3 (nearly every day). A total score of 3 and above indicates that a major depressive disorder is likely.

^d^A total score of 4 or greater is considered positive for loneliness, with scores of 7-9 considered to indicate severe loneliness.

^e^GAD-7: 7-item Generalized Anxiety and Depression scale.

**Table 2:** Clinical outcomes.^a^

Outcome			Participants, n		Baseline, mean (SD)		Follow-up, mean (SD)		Mean change		Percent change
**UCLA-3 Loneliness scores**											
	All	243		7.9 (1.4)		7 (1.9)		0.9		11.1	
	Baseline+30 days	113		7.9 (1.2)		7 (1.9)		0.9		11.8	
	Baseline+60 days	67		8 (1.4)		7.3 (1.9)		0.7		8.9	
	Baseline+90 days	63		7.8 (1.5)		6.7 (1.7)		1.2		14.9	
**PHQ-2^b^** **Depression Scale scores**											
	All	242		3.9 (1.8)		2.8 (1.9)		1.1		28.3	
	Baseline+30 days	112		3.7 (1.8)		2.8 (2.1)		0.9		23.7	
	Baseline+60 days	67		4.3 (1.6)		3.2 (1.9)		1.1		25.4	
	Baseline+90 days	63		3.8 (2)		2.4 (1.9)		1.3		35.2	
Mentally unhealthy days (baseline+90 days)			60	20.8 (10)			15.4 (9.6)		5.5		26.2

^a^This table presents the data of participants who completed optional surveys at 30, 60, and 90 days. All comparisons from baseline to 30, 60, and 90 days were statistically significant at *P*<.001. Engagement rates (ongoing participation with peer support) were 84.4% in month 2 and 70.6% in month 3.

^b^PHQ-2: 2-item Patient Health Questionnaire.

## Discussion

Digital peer support can effectively engage socioeconomically vulnerable populations with significant depression, anxiety, and loneliness across age, gender, and racial/ethnic groups. The 71% engagement rate in the third month was higher than the average 3.3% retention rate at 30 days for digital mental health interventions [10]. Participation was associated with clinically and statistically significant improvements in mental health symptoms and loneliness within 90 days.

The analysis was limited by lack of a control group and brevity of the mental health measures. Future studies should use comprehensive mental health assessments. Additionally, the promising cost savings estimate should be directly measured.

These results suggest that digitally delivered peer support can effectively engage and improve the mental health of vulnerable, ethnically, and sociodemographically diverse populations with symptomatic mental illness and potentially reduce overall medical costs.

## Abbreviations

GAD-7: 7-item General Anxiety Disorder scale
PHQ-2: 2-item Patient Health Questionnaire

## Data Availability

Data included in these analyses will be made available upon reasonable request to the corresponding author.
